# Additive Effects of Mechanical Marrow Ablation and PTH Treatment on *de Novo* Bone Formation in Mature Adult Rats 

**DOI:** 10.3390/cells1041168

**Published:** 2012-12-05

**Authors:** Qing Zhang, Christopher Miller, Jesse Bible, Jiliang Li, Xiaoqing Xu, Nozer Mehta, James Gilligan, Agnès Vignery, Jodi A Carlson Scholz

**Affiliations:** 1 Department of Orthopaedics, Yale University School of Medicine, 310 Cedar Street, New Haven 06510, CT, USA; E-Mails: qing.zhang@yale.edu (Q.Z.); xqing011@yahoo.com (X.X.); agnes.vignery@yale.edu (A.V.); 2 Department of Cell Biology, Yale University School of Medicine, 310 Cedar Street, New Haven 06510, CT, USA; 3 Department of Orthopaedics and Rehabilitation, Yale University School of Medicine, 310 Cedar Street, New Haven 06510, CT, USA; E-Mails: christopher.p.miller@yale.edu (C.M.); jesse.bible@yale.edu (J.B.); 4 Department of Biology, Indiana University Purdue University Indianapolis, 723 West Michigan Street, SL306, Indianapolis 46202, IN, USA; E-Mail: jilili@iupui.edu; 5 Unigene Laboratories, Inc. 81 Fulton Street, Boonton 07005, NJ, USA; E-Mails: nmehta@unigene.com (N.M.); JGilligan@tarsatherapeutics.com (J.G.); 6 Section of Comparative Medicine, Yale University School of Medicine, 310 Cedar Street, New Haven 06510, CT, USA

**Keywords:** PTH, mechanical marrow ablation, bone engineering, aging, site-directed bone formation

## Abstract

Mechanical ablation of bone marrow in young rats induces rapid but transient bone growth, which can be enhanced and maintained for three weeks by the administration of parathyroid hormone (PTH). Additionally, marrow ablation, followed by PTH treatment for three months leads to increased cortical thickness. In this study, we sought to determine whether PTH enhances bone formation after marrow ablation in aged rats. Aged rats underwent unilateral femoral marrow ablation and treatment with PTH or vehicle for four weeks. Both femurs from each rat were analyzed by X-ray and pQCT, then analyzed either by microCT, histology or biomechanical testing. Marrow ablation alone induced transient bone formation of low abundance that persisted over four weeks, while marrow ablation followed by PTH induced bone formation of high abundance that also persisted over four weeks. Our data confirms that the osteo-inducive effect of marrow ablation and the additive effect of marrow ablation, followed by PTH, occurs in aged rats. Our observations open new avenues of investigations in the field of tissue regeneration. Local marrow ablation, in conjunction with an anabolic agent, might provide a new platform for rapid site-directed bone growth in areas of high bone loss, such as in the hip and wrist, which are subject to fracture.

## 1. Introduction

Bone is controlled by the concerted activity of osteoblasts, which synthesize the bone matrix and control its calcification; and by osteoclasts, which resorb bone. Osteoblasts and osteoclasts continuously remodel bone to maintain its mass and define its structure; therefore, these two cells are potential targets for pharmacotherapy, for the prevention and treatment of osteoporosis. Most therapies for osteoporosis that have been developed over the past 20 years target osteoclasts, decreasing their resorptive activity. In contrast, administration of a parathyroid hormone (PTH) induces an anabolic response in bone. First reported nearly 30 years ago by Reeve *et al.* [1], the anabolic effects of PTH have been well characterized and administration of PTH is approved by the Food and Drug Administration in the US for the treatment of osteoporosis. However, it must be injected daily for nearly 18 months to induce an increase in bone mass and a marked reduction in skeletal fractures. For patients with established osteoporosis, it may be desirable to have a more rapid method of fracture prevention at sites that are most affected by osteoporosis, such as the spine, the hip and the forearm. 

We previously reported an additive and rapid effect of mechanical bone marrow ablation in conjunction with daily treatment with PTH on the formation of new bone in the medullary cavity of the femoral shafts of young adult rats [2]. This new bone forms without a cartilage enlagen and is therefore intramembranous. We next reported that extending the daily PTH treatment for three months after marrow ablation leads to a dramatic increase in cortical thickness that persists even after the intramedullary bone is resorbed [3]. Together, these findings supported the possible use of this method for rapid, site-specific bone growth, with specific application for supporting bone grown in anatomical areas particularly prone to osteoporosis-induced fracture in high-risk individuals. However, these studies were conducted in young adult animals, where growth rate may have potentiated the response to marrow ablation. Because osteoporosis is primarily a disease of the aging population, we sought to determine in the current study whether this same response would occur in aging rats. In rats, as with humans, bone metabolism is known to decrease with age; therefore, the mature rat is a suitable model [4,5,6]. While PTH was expected to have a potent anabolic effect in older rats [7,8,9], the response to marrow ablation was not predicted as clearly. Though previous studies on older rats would suggest that marrow ablation would be less osteo-inductive than in younger animals [6,7], previous analyses of bone marrow ablation in aged mice demonstrated a strong formation response [10]. Therefore, we hypothesized that the aged rats would indeed respond to the ablation in much the same manner as young rats. 

## 2. Results and Discussion

Rats were subjected to mechanical bone marrow ablation (BMX) of the left femur. Following BMX, rats were either euthanized on the day of surgery (baseline group, n = 12) or were administered PTH (n = 12) or phosphate buffered saline (PBS, n = 12) for the duration of the four week experiment. Unoperated rats were used as controls (n = 8). All animals recovered uneventfully from the surgery and no perioperative complications were observed. Animals in all groups grew normally throughout the study (Table 1). Following euthanasia at the designated endpoint, both femurs were excised and imaging by x-ray and pQCT analysis was performed on all femurs. For visualization, both femurs from two randomly selected rats from each group were scanned with a microCT scanner. A subset of rats (n = 4–6) from each group were submitted to histomorphometric analysis or biomechanical testing using the three-point bending test. Serum osteocalcin was measured in all animals.

**Table 1:** Animal body weight. Data are presented as mean ± SD. n = 8 for Control; n = 12 for all other groups.

Group	Body weight (g)	
	Day 1	Day 21
Baseline	506.1 ± 60.4	-
Control	608.3 ± 55.5	631.3 ± 54.4
bmx + PBS	540.0 ± 65.4	573.1 ± 58.7
bmx + PTH	580.0 ± 61.5	602.4 ± 50.4

### 2.1. Imaging Analysis

**Figure 1:**
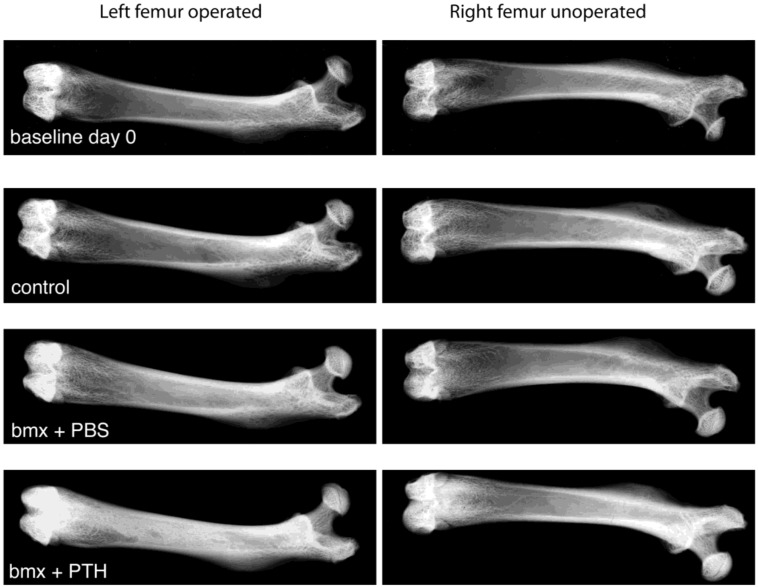
High-resolution radiographs of left operated and right unoperated femurs from baseline, control, and bone marrow-ablated (bmx) rats treated five days per week with phosphate buffered saline (PBS) or parathyroid hormone (PTH) for 28 days. X-ray images are representative of each group. Note the intense radio-opacity of the marrow ablated femur from rats treated with PTH.

**Figure 2:**
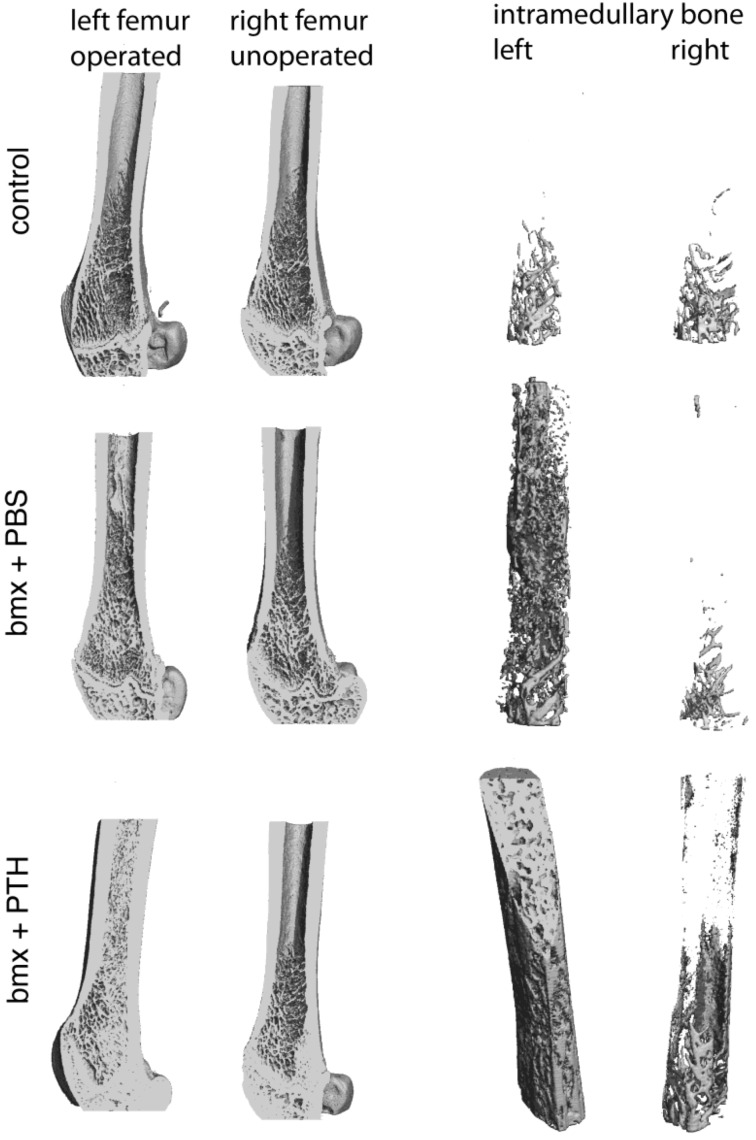
MicroCT analysis of femoral shafts of left operated and right unoperated femurs from control and bone marrow-ablated (bmx) rats treated five days per week with PBS or with PTH for 28 days. The images on the right were generated by “peeling off” the cortical bone to isolate the intramedullary bone. Note the abundance of bone present in lieu of marrow in the ablated femoral shaft from rats treated with PTH.

When subjected to x-ray analysis, ablated femurs from rats treated with PBS revealed mildly enhanced radiopacity compared with control rats, similar to previous results in younger animals [2,3]. In contrast, ablated femurs from rats treated with PTH revealed a highly intense radiopacity (Figure 1). These observations were confirmed by microCT analysis (Figure 2) where “pealing” of cortical bone illustrated the abundance of the new bone formed in the marrow cavity in response to marrow ablation and treatment with PTH. In non-ablated femurs, PTH administration resulted in a mildly enhanced radiopacity (Figure 1) and in a moderate increase in intramedullary bone (Figure 2) compared with PBS administration. To quantify the effects observed, we subjected both femurs from each rat to pQCT analysis. pQCT analysis revealed the additive effect of PTH and marrow ablation on the formation of new bone in these older rats. In rats treated with PTH, total bone density was higher in ablated shafts than in: (i) contralateral non operated shafts; (ii) ablated shafts of rats that received PBS, and (iii) shafts of control rats (Table 2). 

### 2.2. Biomechanical Testing

**Figure 3:**
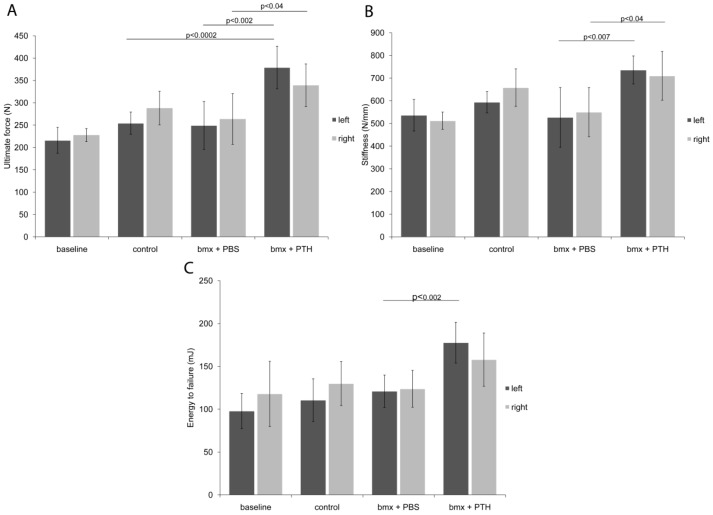
Biomechanical testing results from analysis of femoral shafts of left operated and right unoperated femurs from baseline, control and bone marrow-ablated (bmx) rats treated five days per week with PBS or PTH for 28 days. Right and left femurs were subjected to three-point bending to record (**a**) the ultimate force, (**b**) the energy to failure of femurs, and (**c**) the stiffness. PTH treatment improves mechanical properties of femoral shafts and augments the energy to failure when combined with marrow ablation. (n = 4–6 per group).

To evaluate whether the new bone formed in response to marrow ablation and PTH treatment in these mature adult rats endowed femurs with improved mechanical properties, we subjected both femurs from a subset of rats from each group to three-point bending. PTH treatment not only increased the ultimate strength and the stiffness in the right unoperated femurs (Figure 3B,C), but also potentiated the effects of marrow ablation as it augmented the energy to failure in the operated femurs, an improvement that had not been attained in previous studies on younger rats [2] (Figure 3B). As the cross sectional area did not differ between the PTH-treated marrow ablated group and the PBS-treated marrow ablated group, the enhanced biomechanical properties in the PTH-treated marrow ablated group can be attributed to the newly formed intramedullary bone. Unlike the right, unoperated femurs, energy to failure of the left operated femurs was also significantly increased by PTH treatment when compared with PBS. Although the relative increase in total bone density of the femoral shafts was comparable to what we reported in younger animals, and this in the context of an extra week of PTH treatment in old rats, such an apparent improvement in biomechanical properties could have resulted from the larger diameter of the shafts, since the periosteal perimeter was nearly 50% longer in old rats when compared to young animals. These data suggest that new bone induced by the combined marrow ablation and PTH treatment can increase further bone resistance to bending than PTH alone. 

**Table 2:** Total content, density and area of right and left femurs as measured by pQCT. Data are presented as mean ± SD. a = *p* < 0.001 *vs.* Baseline; b = *p* < 0.001 *vs.* Control; c = *p* < 0.001 *vs.* Right femur; d = *p* < 0.001 *vs.* bmx + PTH. n = 8 for Control; n = 12 for all other groups.

	Group	Total		
		Content (mg)	Density (mg/cm^3^)	Area (mm^2^)
Left femur	Baseline	12.8 ± 1.3	934.5 ± 78.5	13.8 ± 2.0
	Control	16.4 ± 1.9 ^a^	973.1 ± 42.1 ^a^	16.8 ± 1.9 ^a^
	bmx + PBS	15.2 ± 2.2	996.0 ± 62.0	15.2 ± 1.7
	bmx + PTH	18.9 ± 2.5 ^bcd^	1166.2 ± 68.5 ^bcd^	16.2 ± 1.8
Right femur	Baseline	13.0 ± 1.3	936.1 ± 80.9	14.0 ± 2.1
	Control	16.2 ± 1.9	964.3 ± 45.2	16.8 ± 1.8
	bmx + PBS	14.6 ± 1.9	973.5 ± 57.5	15.0 ± 1.6
	bmx + PTH	16.3 ± 2.4	1013.5 ± 75.1	16.0 ± 1.6

### 2.3. Histomorphometry

**Figure 4:**
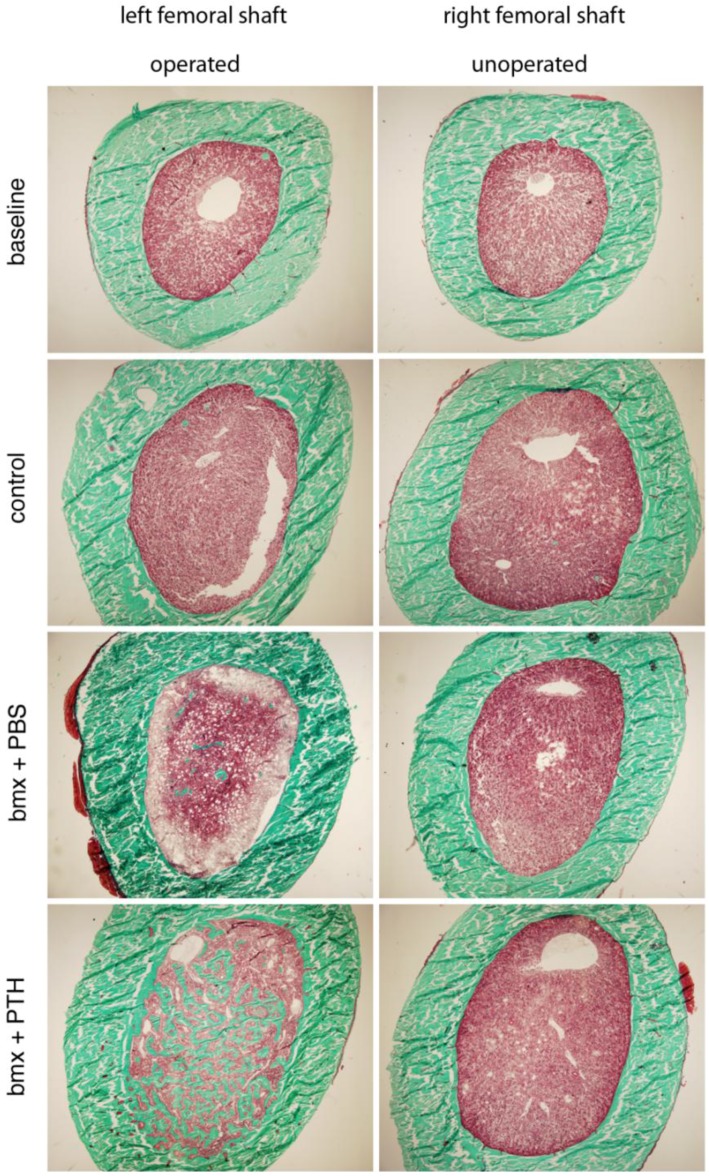
Histologic cross-sections of femoral shafts of left operated and right unoperated femurs from baseline, control and bone marrow-ablated (bmx) rats treated five days per week with PBS or PTH for 28 days. Note the abundant new bone formation in ablated femurs from rats treated with PTH. Mason-Goldner trichrome stain.

**Figure 5:**
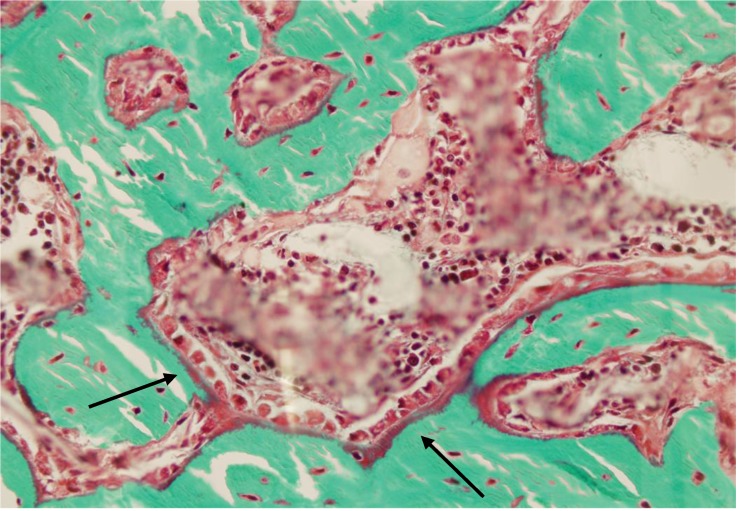
High magnification view of left operated femur a bone marrow-ablated (bmx) rat treated five days per week with PTH for 28 days. Note the presence of active osteoblasts lining the new bone (arrows).

**Figure 6:**
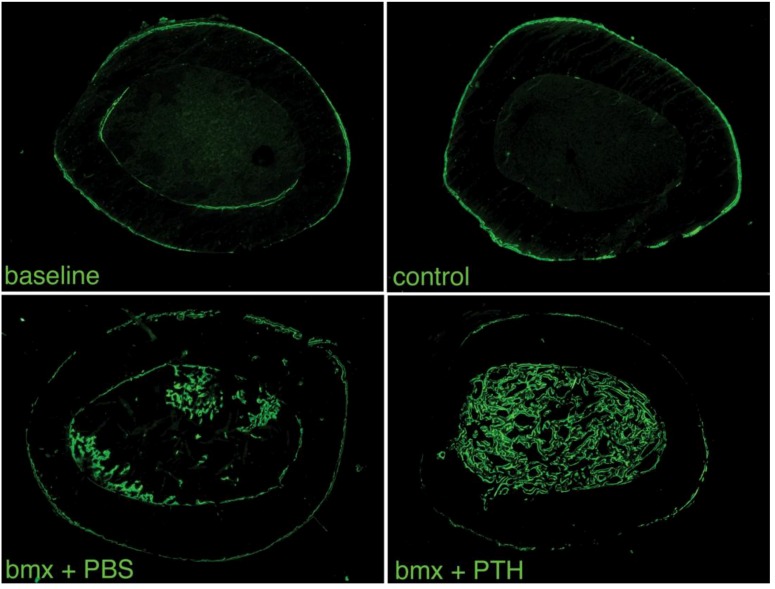
Cross sections of femoral shafts of left operated femurs from baseline, control and bone marrow-ablated (bmx) rats treated five days per week with PBS or PTH for 28 days. The intramedullary bone formed in response to marrow ablation followed by treatment with PTH is undergoing calcification, as demonstrated by the intense fluorescent signal viewed under UV light.

To appreciate the morphological quality of the new bone formed in the marrow cavity, we subjected both femurs from a subset of rats from each group to histological analysis. We observed that, as in younger rats, the woven bone formed in response to marrow ablation was relatively sparse four weeks after surgery in animals that had received PBS. This suggested that the new bone formed had, for the most part, been resorbed by osteoclasts. In contrast, the new bone formed in ablated femurs from rats treated with PTH was extremely abundant, to an extent that appeared similar to that reported previously in younger rats (Figure 4) [2,3]. Higher magnification analysis of that new bone revealed its lining by active osteoblasts, and the presence of small osteocytic lacunae, indicating that the new bone was being actively formed (Figure 5). In addition, since the rats were given calcein before sacrifice, we were able to appreciate the intense fluorescent signal that lined most of the surface of the new intramedullary bone, which is indicative of ongoing calcification. As in younger rats [2], such a signal was weak and diffuse in the marrow cavity of ablated shafts from rat that had received PBS, and absent in shafts from baseline and control rats (Figure 6). These observations supported an additive bone anabolic effect of marrow ablation and PTH treatment in older rats. 

### 2.4. Serum Osteocalcin

Serum osteocalcin was measured at the time of sacrifice as a biomarker for bone formation. Rats that underwent marrow ablation and were treated with PTH had significantly higher osteocalcin levels compared to control rats and rats that underwent marrow ablation and were treated with PBS (Table 3), indicating active bone formation. Baseline rats also had a higher concentration of osteocalcin than control rats, which may be due, at least in part, to a decline in serum osteocalcin occurring with age [11]. 

**Table 3:** Serum osteocalcin concentration. n = 8 for Control; n = 12 for all other groups.

Group	Osteocalcin (mg/mL)	Level of Significance
Baseline	58.4 ± 9.7	*p* < 0.007 *vs.* Control
Control	44.0 ± 10.9	*p* < 0.0002 *vs.* bmx + PTH; *p* < 0.007 *vs.* Baseline
bmx + PBS	45.9 ± 8.3	*p* < 0.0001 *vs.* bmx + PTH
bmx + PTH	61.8 ± 8.7	*p* < 0.0001 *vs.* PTH; *p* < 0.0002 *vs.* Control

## 3. Experimental

### 3.1. Animals

Retired male breeder Sprague Dawley rats (Crl:SD), approximately nine-months old, were obtained from Charles River (Kingston, NY). All rats were allowed to acclimate for two weeks after arrival at the Yale Animal Resources Center and housed under controlled room temperature (72 ± 2F), humidity (50 ± 20%), and light (12 h dark /12 h light) with food and water available ad libitum. The care and treatment of experimental animals complied with all applicable federal guidelines and was approved by the Institutional Animal Care and Use Committee at Yale University. All rats were free of corona virus, Sendai virus, pneumonia virus of mice, *Mycoplasma*, and ecto- and endoparasites. 

### 3.2. Experimental Design

Rats were divided into four groups: Baseline (n = 8); control (n = 12); bone marrow ablation (bmx) + PBS (n = 12); and bmx + PTH (n = 12). Baseline rats were subjected to bone marrow ablation surgery as described below and were sacrificed immediately after surgery. The bmx + PBS and bmx + PTH groups underwent bone marrow ablation and were subsequently administered either PBS or PTH as described below for 28 days, at which time they were sacrificed. As only one femur was subjected to bone marrow ablation, the contralateral femur served as an unoperated control. Control rats did not undergo any intervention and were sacrificed at the same time as the bmx + PBS and bmx + PTH rats. At the time of sacrifice, rats were deeply anesthetized and blood was collected by cardiac puncture. Both femurs were removed immediately post mortem and processed for analysis as described below. 

### 3.3. Femoral Bone Marrow Ablation Procedure

Bone marrow ablation was performed as previously described [2,3]. In brief, rats were anesthetized with a combination of ketamine (50 mg/kg) and xylazine (10 mg/kg) intraperitoneally. Hair over the left knee joint was shaved and the area was prepared with betadine scrub and ethanol. A 1.0-cm-long longitudinal skin incision was made across the medial aspect of the knee joint. The distal femur was exposed by lateral luxation of the patella, which was accomplished by release of the medial ligamentous structures. A 1.0-mm-hole was drilled through the femoral intracondylar notch using a round carbide burr (#2, SS White, Ethical Dental) through the growth plate, into the marrow cavity. An antimicrobial bristle brush (Product # 45-542); 2 mm diameter, 12 mm long, Sharn, Inc., Tampa, FL, USA) was used to remove cells and debris from the bone marrow cavity, and the marrow was then back-flushed by injection of 5 ml of normal saline solution into the femur using a syringe attached to a 21-gauge needle. This process removed the bone marrow cells while keeping the bone structures intact, except for the defect created by the drill in the distal cortex and growth plate. The medial ligamentous structures were sutured with a 4-0 Vicryl. The skin incision was closed with surgical metallic clips. The rats were injected subcutaneously (s.c.) with a 5-ml bolus of saline and were given carprofen (5 mg/kg/day orally) for the first 48 h after surgery. 

### 3.4. PTH or PBS Administration

A recombinant analog of human PTH (PTH1-34 NH2) was provided by Unigene Laboratories, Inc. (Boonton, NJ, USA) [12]. PTH (40 μg/kg/day) and PBS (volume equal to PTH) were injected s.c. in the dorsal neck region of the animals. Injections were initiated on the day of surgery (day 1) and were performed for 5 consecutive days per week for 4 weeks. 

### 3.5. Bone Radiography

Both excised femurs from each rat were subjected to x-ray on a cranial-caudal view using a Kubtec unit (Fairfield, CT, USA) at 30 kV for four seconds. X-ray films were scanned using an Epson Perfection 4870. 

### 3.6. Bone Densitometry by Peripheral Quantitative Computed Tomography (pQCT)

Bone density was determined for both femurs in each rat as we described previously [13] by pQCT with a Stratec scanner (model XCT Research; Norland Medical Systems, Fort Atkinson, WI). Routine calibration was performed daily with a defined standard that contained hydroxyapatite crystals embedded in lucite, provided by Norland Medical Systems. One mm thick slices were made at a location midway between epiphyses, at the center of the femoral shaft. The voxel size was set at 0.1 mm. Scans were analyzed with a software program supplied by the manufacturer (XCT 520, version 5.1). Bone density and geometric parameters were estimated by Loop analysis. The low- and high-density threshold settings were 1,300 and 2,000, respectively. Separation of soft tissue from the outer edge of bone was achieved using contour mode 1. 

### 3.7. Computed Tomography on a Microscale (microCT)

Both femurs from two randomly selected rats from each group were scanned with a microCT scanner (MicroCT 40; Scanco, Bassersdorf, Switzerland) with a 2,048 × 2,048 matrix and isotropic resolution of 9 μm^3^ (12 μm voxel size). Three dimensional trabecular measurements in the medullary cavity were made directly. 

### 3.8. Biomechanical Testing of the Femoral Midshaft: Three-Point Bending Test

Right and left femurs from 4–6 rats per group were subjected to three-point bending to record the ultimate force, the stiffness and the energy to failure of femurs as we previously published [2]. The anterior to posterior diameter at the midpoint of the femoral shaft was recorded using an electronic caliper. Femurs were placed on the lower supports of a three-point bending fixture with the anterior side facing downward in an Instron Mechanical Testing Instrument (Norwood, MA, USA; Instron 4465 retro fitted to 5500). The span between the two lower supports was set at 14 mm. The upper loading device was aligned to the center of the femoral shaft. The load was applied at a constant displacement rate of 6 mm/min until the femur broke. The locations of maximal load, stiffness and energy absorbed were selected manually from the load. 

### 3.9. Histology

Femurs from 4-6 randomly chosen rats per group were dehydrated in a graded ethanol series and embedded without decalcification in methyl methacrylate, as we described previously [2,3]. Transversal 4-8 micron-thick sections were obtained the femoral shafts, mid-way from the epiphyses, using an Autocut^TM^ microtome equipped with a tungsten carbide blade (RM 2265; Leica, Germany). Sections were kept unstained or stained with Mason-Goldner trichrome. To evaluate active bone formation, rats received four s.c. injections of calcein (10 mg/g body weight; Merck, Darmstadt, Germany) on days 14, 13, 4 and 3 before sacrifice and unstained sections were viewed with epifluorescence illumination for histological analysis as we previously described [14,15]. Despite the excellent quality of the histologic sections, the accuracy of static or dynamic histomorphometric analysis was hindered by the severe heterogeneity of the bone structure and the density and variety of cells present in the marrow, which occurs after marrow ablation; therefore, detailed histomorphic analysis was not performed. 

### 3.10. Microscopy

Microscopy was performed using an IMT-2 Olympus microscope equipped with ultraviolet light (UV) and an OM-4 camera. 

### 3.11. Biochemical Parameters

Blood was collected by cardiac puncture at the time of sacrifice and the concentration of serum osteocalcin was determined as described previously [16]. Briefly, osteocalcin (Ocn) levels in the sera were measured in-house by a standard equilibrium RIA. 

### 3.12. Statistical Analysis

Data represent the mean ± one standard deviation (SD). Treatment groups were compared using the analysis of variance. Statistical significance was declared if the two-sided p-value was <0.05. 

## 4. Conclusions

While we had previously reported the additive effect of mechanical marrow ablation and treatment with PTH on the targeted formation of new intramedullary bone, both previous studies were performed in young adult animals and therefore left open the possibly that the formation of new bone was potentiated by the fast growth rate of young rodents [2,3]. Here we report that rapid and abundant bone formation in response to marrow ablation and PTH treatment also occurs in nine-month old rats. Because mechanical ablation of bone marrow is followed by the rapid and transient formation of new bone in mice [17,18,19,20] and rabbits (Carlson Scholz *et al.*, unpublished data) in addition to rats, it is possible that aspiration of bone marrow at sites that are at risk for fracture, such as the hip, might lead to the transient formation of new bone in lieu of marrow in humans. It is tempting to further predict that PTH combined with marrow aspiration might promote rapid and long lasting bone formation in humans, and/or increase cortical bone density in humans, which PTH treatment alone fails to achieve [21,22]. While the treatment of osteoporotic women with recombinant human parathyroid hormone (rhPTH [1-34]) reduces fracture risk, the gains in bone mineral density require several months during which time fractures can occur. It is our hope that future studies will address whether targeting of new bone to specific skeletal sites can be applied to humans, and can achieve efficacy in fracture prevention. In addition, deciphering the circuitry of genes regulated in response to PTH treatment following marrow ablation would help improve our understanding of the molecular mechanisms that initiate and promote intra membranous bone formation [23].
